# Anti-Atherosclerosis Effect of Angong Niuhuang Pill *via* Regulating Th17/Treg Immune Balance and Inhibiting Chronic Inflammatory on ApoE^-/-^ Mice Model of Early and Mid-Term Atherosclerosis

**DOI:** 10.3389/fphar.2019.01584

**Published:** 2020-01-31

**Authors:** Qinghong Fan, Yujuan Liu, Jiaoyu Rao, Zhe Zhang, Wei Xiao, Tao Zhu, Xiaomeng Chai, Kaihe Ye, Na Ning, Zhen Yin, Yushuang Chai, Yimin Xu, Ruirui Lan, A Verkhratsky, Hong Nie

**Affiliations:** ^1^ Guangdong Province Key Laboratory of Pharmacodynamic Constituents of Traditional Chinese Medicine and New Drugs Research, College of Pharmacy, Jinan University, Guangzhou, China; ^2^ Guangzhou Baiyunshan Zhongyi Pharmaceutical Co., Ltd, Guangzhou, China; ^3^ International Department, The Affiliated High School of SCNU, Guangzhou, China; ^4^ Faculty of Biology, Medicine and Health, The University of Manchester, Manchester, United Kingdom; ^5^ International Cooperative Laboratory of Traditional Chinese Medicine Modernization and Innovative Drug Development of Chinese Ministry of Education (MOE), College of Pharmacy, Jinan University, Guangzhou, China

**Keywords:** Angong Niuhuang Pill, early and mid-term atherosclerosis, ApoE^-/-^ mice, Th17/Treg balance, inflammation, plaque stability

## Abstract

Angong Niuhuang Pill (ANP) is a well-known patented Chinese medicine which is used for hundreds of years for treating the central nervous system diseases. Atherosclerosis is a poly-aetiological chronic inflammatory vascular disease. Preventing inflammation is fundamental for treating atherosclerosis in early stages. In this study, we investigated the protective effects and possible mechanisms of ANP action on a high-fat diet induced early and mid-term atherosclerosis ApoE^-/-^ mice. The effects of ANP were compared with accepted drug simvastatin. Twelve male C57BL/6J mice were used as the control group, and 60 male ApoE^-/-^ mice were randomly divided into five groups: Model group, Simvastatin group, Low-, Medium-, and High-dose ANP group these groups received, respectively, saline, simvastatin (3.0mg/kg), low-dose ANP (0.25 g/kg), medium-dose ANP (0.50 g/kg), and high-dose ANP (1.0 g/kg), once every other day for 10 weeks. After administration, serum biochemical indices were detected by the automatic biochemical analyzer, the concentrations of IL-6 and IL-10 in the serum were assayed by ELISA, expression levels of IL-1β, TNF-α, MMP-2, MMP-9, CCL2, and its receptor CCR2 in the full-length aorta, and expression levels of transcription factors Foxp3, RORγt in the spleen were assayed *via* western blotting and RT-qPCR. Flow cytometry was used to analyze Th17 cells and Treg cells. Pathological and histological analysis was completed on aortic root. ANP decreased LDL/HDL ratio, concentrations of IL-6 while increased IL-10 in serum. Moreover, ANP down-regulated the expression levels of IL-1β, TNF-α, MMP-2, MMP-9, CCL2, and CCR2 receptor in the full-length aorta. In addition, ANP decreased Th17 cells and expression levels of transcription factor RORγt, increased Treg cells and expression levels of transcription factor Foxp3. ANP decreased content of collagen fibers and infiltration of inflammatory cells in the aortic root. In conclusion, we demonstrated that ANP has anti-atherosclerosis effects on a high-fat diet induced ApoE^-/-^ mice early and mid-term AS model *via* regulating Th17/Treg balance, inhibiting chronic inflammation, reducing plaque collagen fibers, and reducing inflammatory cells infiltration, to exert its multi-channel multi-target anti-early and mid-term AS effects.

## Introduction

Cardiovascular diseases (CVD) and related chronic diseases are the number one cause of death. About 80% of CVD-related deaths occur in low-income and middle-income countries (Feigin et al., 2015). According to China Cardiovascular Disease Report 2017, the number of patients with CVD reached 290 million, and CVD deaths account for more than 40% of deaths. Since 2004, the average growth rate of CVD cost in China was much higher than the growth rate of GDP (Chen et al., 2018). The burden of China’s CVD is increasing; it has become a major public health problem. It is well accepted that aterosclerosis (AS) is the primary cause of CVDs. Therefore, the search for effective therapy is of fundamental importance.

Pathogenesis of AS includes lipid infiltration, damage response, mononuclear macrophage invasion, and inflammation response. At present, it is generally accepted that AS is a chronic inflammatory disease (Ross, 1999), which involves cellular immune response, particularly of Th17 cells and CD4^+^CD25^+^ regulatory T (Treg) cells (Xie et al., 2010; Wu et al., 2017). Treg and Th17 cells have recently been described as two distinct subsets distinct from Th1 and Th2 cells, while Th17/Treg balance is different from balance of Th1/Th2 (Frostegard et al., 1999; Cheng et al., 2008). Treg cells expressing the forkhead/winged helix transcription factor (Foxp3) and Th17 cells expressing retinoic acid-related orphan receptorγt (RORγt) are two important immune cells for controlling anti-atherogenic cytokines such as interleukin (IL)-10, transforming growth factor (TGF)-β1, and pro-atherogenic cytokines such as IL-6, IL-17 (Shimon et al., 2006; Bettelli et al., 2007). Above cytokines play important roles in maintaining the number and function of Treg cells and Th17 cells and participate in the pathogenesis of human AS. In previous study (Chai et al., 2019), we reported that the Th17/Treg imbalance existed in the ApoE^-/-^ mice AS model, which was induced by consecutive 8 weeks high-fat diet. Therefore, the Th17/Treg balance is critical for prevention of AS and autoimmune response.

Chronic inflammation represents the most basic pathogenetic factor of AS (Ross, 1999), Cytokines orchestrate the complex inflammatory response within the atherosclerotic plaque throughout the entire AS evolution (Tousoulis et al., 2016). At the early and middle stages of AS, endothelial dysfunction, secretion of chemokines and cytokines such as CCL2, IL-1β, TNF-α expression of intercellular adhesion molecule 1 (ICAM-1), vascular cell adhesion molecule 1 (VCAM-1), accumulation of immune cells such as T lymphocytes, dendritic cells, macrophages to the AS plaques, and migration and proliferation of vascular smooth muscle cells (VSMCs) to these plaques further aggravate inflammatory response (Ramji and Davies, 2015). Furthermore, activation of matrix metalloproteinases (MMPs) instigates expansion and instability of atherosclerotic plaques (Ramji and Davies, 2015; Tousoulis et al., 2016). The expanding plaque once ruptured may cause arterial thrombosis, stenosis or blockage of the lumen, affecting the arterial supply of blood to organs and tissues, eventually leading to cerebral infarction, acute coronary syndrome, myocardial infarction, and peripheral vascular disease (Weber and Noels, 2011). In the previous study (Chai et al., 2019), we found that ApoE^-/-^ mice induced by 8 weeks high-fat diet demonstrate severe inflammatory response in the full-length aorta. Therefore, inhibiting this inflammatory response, reducing migration and adhesion of inflammatory cells, together with maintaining plaque stability are essential for the treatment of early and mid-stage AS.

Angong Niuhuang Pill (ANP) is a well-known patented Chinese medicine, used to treat stroke, encephalitis, and meningitis. Its main components include *Bovis Calculus*, *Powerdered Buffalo Horn Extract*, Artificial *Moschus*, *Cinnabaris*, *Coptis chinensis* Franch., *Hyriopsis cumingii*(Lea), *Scutellaria baicalensis* Georgi, *Realgar*, *Gardenia jasminoides* J.Ellis, *Curcuma aromatica* Salisb., and *Borneolum Syntheticum* (Editorial Committee of Pharmacopoeia of Ministry of Health PR China, 2015). The chemical structure of the known component in artificial Moschus were shown in **Supplementary Material (Table 1)**. Pharmacological effects of ANP include: hepatoprotection, anti-inflammation, anti-viral action, antipyresis, and anticonvulsive effects. Our previous study also found that ANP has anti-atherosclerosis and cardio-protective effect on high-fat diet combined with vitamin D3 induced AS rats (Fu et al., 2017). In the present study, we combine the studies of Th17/Treg balance and inflammatory pathways to attempt to investigate the possible mechanisms of ANP relevant for treating the early and middle AS in the ApoE^-/-^ mice induced by 10-week high-fat diet (Nakashima et al., 1994; Gupta et al., 1997; Meir and Leitersdorf, 2004).

## Materials and Methods

### Preparation of ANP

ANP was provided by Guangzhou Baiyunshan Zhongyi pharmaceutical co., Ltd (Guangzhou, China). It contains the following components: *Bovis Calculus* 100 g, *Powerdered Buffalo Horn Extract* 200 g, Artificial *Moschus* 25 g, *Hyriopsis cumingii* (Lea) 50 g, *Cinnabaris* 100g, *Realgar* 100 g, *Coptis chinensis* Franch. 100 g, *Scutellaria baicalensis* Georgi 100 g, *Gardenia jasminoides* J. Ellis 100 g, *Curcuma aromatica* Salisb. 100 g, *Borneolum Syntheticum* 25 g (Editorial Committee of Pharmacopoeia of Ministry of Health PR China, 2015).

*Cinnabaris*, *Hyriopsis cumi-ngii* (Lea) and *Realg*ar were grounded or pulverized to the very fine powders (200 mesh). *Coptis chinensis* Franch., *Scutellaria baicalensis* Georgi, *Gardenia jasminoides* J. Ellis, and *Curcuma aromatica* Salisb. were pulverized to a fine powder (100 mesh). *Bovis Calculus*, *Powerdered Buffalo Horn Extract*, Artificial *Moschus,* and *Powerdered Buffalo Horn Extract* were triturated with the above powders, sifted and well mixed. Refined honey was mixed to make 600 big honeyed pills, or alternately coated with a gold film (Editorial Committee of Pharmacopoeia of Ministry of Health PR China, 2015). HLPC was used to verify the formulation to guarantee the quality of the ANP, fingerprint of ANP please see the **Supplementary Figure S1**. The UPLC Fingerprint of 24 ANP Batches was shown in **Supplementary Figure S3**. 

### Reagents

Main reagents used in the study were as follows: 1) ANP (Guangzhou Baiyunshan Zhongyi Pharmaceutical Co, Ltd, Lot: WA0076); 2) Simvastatin (Shangdong Xinqi Pharmaceutical Co., Ltd, Lot: 20170101); 3) High fat feed composed of 3% cholesterol, 0.5% sodium cholate, 0.2% propylthiouracil, 5% sugar, 10% lard, and 81.3% basic feed (Medical Science Experimental Animal Center, Guangdong, China, Lot: 201814); 4) Kits for IL-6, IL-10 (Wuhan Huamei Biological Co, Ltd, Lot: U23013173, U24018179), kit for reverse transcription, fluorescent dyes of SYBR (TAKARA, Lot: AI12361A, AK9304), kit for Masson staining (Sinopharm Chemical Reagent Co, Ltd, Lot: G1006), kit for BCA (Shanghai Beyotime biotechnology, Lot: 051018180514), and kit for ECL plus (Applygen, Lot: P1010); 5) Anti-αSMA, anti-CD11c, anti-CD68, fluorescent secondary antibody with FITC marker, and fluorescent secondary antibody with CY3 marker (Servicebio, Lot: GB130441, GB11058, GB11067, GB223011, GB21303); 6) β-actin, anti-MCP-1 (CST, Lot: 15,2), anti-CCR2, anti-Foxp3, anti-IL-1β, anti-TNF-α (Abcam, Lot: GR286626-10, GR239541-14, GR309542-2, GR235155-19), anti-RORγ (Santa, Lot: F3017), anti-MMP-2, and anti-MMP-9 (Wanleibio, Lot: WL03334, WL02141); 7) Horseradish peroxidase-conjugated goat anti-rabbit secondary antibody, horseradish peroxidase-conjugated goat anti-mouse secondary antibody (Shanghai Beyotime biotechnology, Lot: 040818180510, 040818184521); 8) PE anti-mouse CD25, APC anti-mouse CD127, PE/Cy7 anti-mouse CD4, Alexa Fluor^®^ 488 anti-mouse IL-17A, APC Rat IgG2a κ Isotype Ctrl, PE Rat IgG2b κ Isotype Ctrl, Alexa Fluor^®^ 488 Rat IgG1 κ Isotype Ctrl, PE/Cy7 Rat IgG2a κ Isotype Ctrl, Cell Activation Cocktail with Brefeldin A, 10×RBC Lysis Buffer, Fixation Buffer, 10×Intracellular Staining Permeabilization Wash Buffer (Biolegad, Lot: B247733, B232775, B249463, B259048, B238057, B255375, B236504, B236195, B248910, B250015, B252968, B252856).

### Animals

Twelve 4-week old Specific Pathogen Free (SPF) male C57BL/6J mice (weight:18 ± 2.0g) and sixty 4-week old SPF male ApoE^-/-^ mice (weight:18 ± 2.0g), were provided by the department of Laboratory Animal Science of the department of medicine, Peking University [Certificate No. SCXK (jing) 2011-0012]. Mice were housed in the Jinan University Medical School Laboratory Animal Management Center [Certificate No. SCXK (Guangdong) 2012-0117] and were maintained at 24°C and 65% humidity. Mice were maintained on a 12-h light/dark cycle, C57BL/6J mice were given free access to standard laboratory mouse chow and tap water, ApoE^-/-^ mice were given free access to high fat feed and tap water. The experiments were approved by the Laboratory Animal Ethics Committee of Jinan University (No. 201812374), and were performed according to the instructions of the National Institute of Health (OLAW/NIH Revised 2015) (Office of Laboratory Animal Welfare/National Institutes of Health, 2015).

### Model Establishment

After ten days of adaptive feeding, 60 SPF male ApoE^-/-^ mice were randomly divided into five groups, i) model group, ii) simvastatin group (3.0 mg/kg), iii–v) low-, ledium-, and high-dose ANP groups (0.25 g/kg, 0.50 g/kg, 1.0g/kg, respectively) and given free access to high fat feed and tap water for 10 weeks. Each group contained twelve mice. Twelve SPF male C57BL/6J mice were used as controls and were given free access to standard laboratory mouse chow and tap water for 10 weeks. On the first day of high-fat diet administration, all experimental drug treatments (simvastatin and ANP) were administered intragastrically once every other day for 10 weeks. Drug dosages were titrated according to animal weight. Saline was given intragastrically in control group and model group. All drug treatments were prepared immediately before administration as a suspension by dissolving an appropriate amount of the drug in distilled water.

### Sample Collection

After administration, serum was collected for biochemical analysis. The aortic root was separated for immunofluorescence staining and Masson staining. The full-length aorta was separated for Real-time quantitative polymerase chain reaction (RT-qPCR) and Western Blotting analysis. The liver, kidney, spleen, and thymus were separated for organ coefficient or organ index. Primary splenocytes were separated for flow cytometric analysis from the spleen.

### Serum Biochemical Analysis

The cholesterol (CHOL), triglyceride (TG), low-density lipoprotein (LDL-C), and high-density lipoprotein (HDL-C) were measured using automatic biochemical analyzer. All protocols were followed in accordance to the manufacturer recommendations.

### Organ Coefficient and Index Analysis

Liver coefficient, kidney coefficient, thymus index, and spleen index were calculated according to corresponded calculation formula separately. The formula of liver and kidney coefficient: weight of liver and kidney (mg)/weight of animal (g). The formula of thymus and spleen index: weight of thymus and spleen (mg)/weight of animal (g)*10.

### Flow Cytometric Analysis of Th17 and Treg Cells

The ratio of Th17 to Treg cells in splenocyte suspensions was analyzed by flow cytometry. The splenocyte suspensions were stimulated with 10 μl of Cell Activation Cocktail (with Brefeldin A) and mixed. After incubation at 37°C for 6 h, the sample was centrifuged, and the precipitate was suspended in 1 ml RBC. The suspensions were then centrifuged and the precipitate re-suspended in PBS twice. After antibody labeling of surface proteins, the sample was incubated in the dark for 20 min followed by centrifugation (1,500 rpm). The cells in the precipitate were then fixed with fixation buffer, followed by incubation in the dark for 20 min. The sample was again centrifuged and suspended in 1 mL Permeabilization Wash Buffer. The cells were then washed twice and labeled with an immunofluorescent antibody. After incubation in the dark for 20 min, the sample was washed using 1 ml Permeabilization Wash Buffer twice and suspended in PBS prior to flow cytometric detection.

### Masson Staining

Masson staining was used to detect collagen fibers in the plaque of aortic root. According to kit for Masson staining manufacturer instructions, the aortic root fixed in 4% paraformaldehyde were dehydrated in alcohol, paraffin-embedded, sectioned and subjected to Masson staining. The stained sections were observed under the light microscope. Masson staining of the whole aorta was measured by Image Pro Plus 6.0. The area proportion of collagen fiber was calculated as the ratio of stained area to area of interest calculated by Image Pro Plus 6.0.

### Immunofluorescence Staining

Immunofluorescence staining was used to detect inflammatory cells infiltration such as macrophages, DCs, and VSMCs in the plaque of aortic root. Anti-CD68, anti-CD11c, and anti-αSMA were respectively used as special marker in macrophages, DCs, and VSMCs. Frozen slices of aortic root were blocked in blocking buffer for 60 min, aspirated blocking solution, applied diluted primary antibody, incubated overnight at 4°C, rinsed three times in PBS for 5 min each, and then incubated specimen in fluorochrome-conjugated secondary antibody diluted in antibody dilution buffer for 1–2 h at room temperature in the dark, rinsed in PBS, thereafter, coverslips stained with DAPI. The stained sections were observed under fluorescence microscope, and Image J was used to semi-quantitatively analyze positive cells.

### Cytokines Detection by ELISA

The serum concentrations of IL-6 and IL-10 were measured using the IL-6 and IL-10 ELISA kits. In line with the manufacturer’s instructions, standard, or sample (100 µl) was added to each well and incubated for 2 h at 37°C. Then, the medium in each well was discarded, and biotin antibody (100 µl) was added to each well, followed by incubation for 1 h at 37°C. The medium in each well was aspirated and the wells were washed three times. Thereafter, HRP-avidin (100 µl) was added to each well and incubated for 1 h at 37°C. The medium in each well was aspirated and the wells were washed five times, followed by addition of tetramethylbenzidine substrate (90 μl) and incubation for 20 min at 37°C in the dark. Finally, the stop solution (50 ul) was added to each well, and the absorbance of the wells was read at 450 nm within 5 min.

### Real-Time Quantitative Polymerase Chain Reaction

The mRNA levels of *Foxp3, RORγt* in the spleen, as well as *CCR2, CCL2, IL-1β, TNF-α, MMP-2,* and *MMP-9* in the full-length aorta, were analyzed by RT-qPCR. Total RNA was extracted with Trizol reagent according to the manufacturer instructions from full-length aorta and spleen. The concentration and purity of RNA was analyzed using a micro-detector. Reverse transcription kit was used for reverse transcription reaction to synthesize cDNA, RT-qPCR was performed to determine mRNA levels of inflammatory mediators with the SYBR Green real-time PCR master mix kit. Each sample was analyzed in triplicate, normalized to *GAPDH*. All the PCR primers used are listed in **Table 1**. RT-qPCR conditions were 95°C for 30 sec followed by 40 cycles of 95°C for 5 sec, 55°C for 30 sec, and 72°C for 60 sec.

**Table 1:** Primers used for RT-qPCR.

Gene	Primer	Sequence 5′-3′
Foxp3	Forward primer	5’- TGGAACCACGGGCACTATCACA-3’
Foxp3	Reverse primer	5’- GAGGCTGCGTATGATCAGTTATGC-3’
RORγt RORγt	Forward primer Reverse primer	5’- TGCAAGACTCATCGACAAGG-3’ 5’- AGGGGATTCAACATCAGTGC-3’
CCR2	Forward primer	5’- AGAGAGCTGCAGCAAAAAGG-3’
CCR2	Reverse primer	5’- GGAAAGAGGCAGTTGCAAAG-3’
CCL2	Forward primer	5’- AGGTCCCTGTCATGCTTCTG-3’
CCL2	Reverse primer	5’- TCTGGACCCATTCCTTCTTG-3’
IL-1β	Forward primer	5’- ACTCATTGTGGCTGTGGAGA-3’
IL-1β	Reverse primer	5’- TTGTTCATCTCGGAGCCTGT-3’
TNF-α	Forward primer	5’- TCTACTGAACTTCGGGGTGATCG-3’
TNF-α	Reverse primer	5’- ACGTGGGCTACAGGCTTGTCA-3’
MMP-2	Forward primer	5’-CACACCAGGTGAAGGATGTG-3’
MMP-2	Reverse primer	5’-AGGGCTGCATTGCAAATATC-3’
MMP-9	Forward primer	5’-TGAATCAGCTGGCTTTTGTG-3’
MMP-9	Reverse primer	5’-GTGGATAGCTCGGTGGTGTT-3’
GAPDH	Forward primer	5’-CATCCATGACAACTTTGGCA-3’
GAPDH	Reverse primer	5’-CCTGCTTCACCACCTTCTTG-3’

### Western Blotting Analysis

Protein levels of Foxp3, RORγt in the spleen, as well as CCR2, CCL2, IL-1β, TNF -α, MMP-9, and MMP-2 in the full-length aorta were detected by western blotting analysis. Total proteins were extracted from full-length aorta and spleen. The concentrations of protein were determined by BCA protein assay. Each sample containing 30 µg protein was separated, respectively, on 8%, 10%, and 15% SDS-PAGE gel electrophoresis and then transferred onto a polyvinylidene difluoride membrane. Thereafter, membranes were incubated in a 5% (weight in volume) milk solution for 2 h and then incubated at 4°C overnight with one of the following primary antibodies: β-actin, IL-1β, TNF-α, CCL2, CCR2, Foxp3, RORγt, MMP-2 (1:1000 dilution each), and MMP-9 (1:500). After primary antibody incubation, membranes were washed and incubated with horseradish peroxidase-conjugated goat anti-rabbit secondary antibody (1:5000, 374°C, 2h) or horseradish peroxidase-conjugated goat anti-mouse secondary antibody (1:2000, 374°C, 2h). The blots were visualized using enhanced chemiluminescence reagent, and UVP BioSpectrum Imaging System was used to expose immune-positive bands. The bands were semi-quantitatively analyzed with Image J, and the results are expressed as ratios of IL-1β, TNF-α, CCL2, CCR2, Foxp3, RORγt, MMP-2, MMP-9 to β-actin densitometry readings.

### Statistical Analysis

All data were performed as mean ± standard error of the mean (mean±SEM) and were analyzed by One-way ANOVA with Dunnett T3 test in SPSS Statistics 18.0. *p* < 0.05 were considered as statistically significant.

## Results

### Body Weight, Liver and Kidney Coefficients

**Figure 1:**
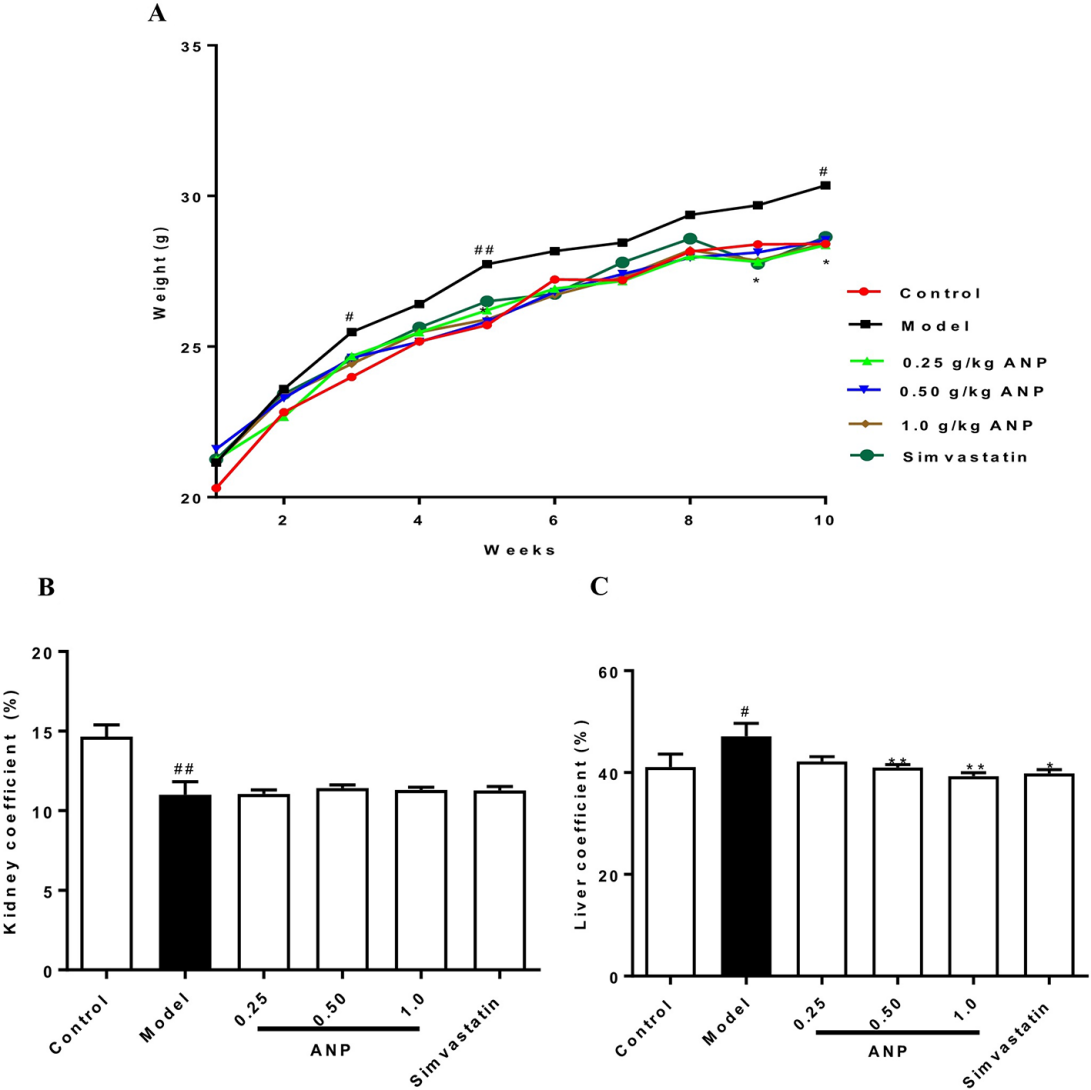
The body weight, liver coefficient and kidney coefficient of ApoE^-/-^ mice and C57BL/6J mice. **(A)** Average body weight curve. **(B)** Kidney coefficient. **(C)** Liver coefficient. Compared with control group, ^#^ *p* < 0.05, ^##^ *p* < 0.01; Compared with model group, *p < 0.05, **p < 0.01 (Mean ± SEM, liver and kidney coefficient n = 6). ANP, Angong Niuhuang Pill.

The body weight of ApoE^-/-^ mice and C57BL/6J mice over a 10-week period was shown in **Figure 1A** and **Table 2**. The body weight of model group was higher than control group, and the body weight of model group was significantly increased at 3^rd^, 5^th^, and 10^th^ week (*p* < 0.05 or *p* < 0.01) compared to the control group. The body weight of ANP groups and simvastatin group was lower than model group, and a significant difference was noted at 5^th^, 9^th^, and 10^th^ week (*p* < 0.05 or *p* < 0.01), compared to the control group. Liver coefficient was significantly increased (*p* < 0.05) while kidney coefficient was significantly decreased (*p* < 0.01) in the model group. In medium- and high-dose ANP groups liver coefficient was significantly decreased (*p* < 0.01). Kidney coefficient was not altered in all ANP groups. These results were shown in**Figure 1B–C**.

**Table 2:** Body weight of C57BL/6 mice and ApoE^-/-^mice in 10 weeks.

Weeks	Groups					
	Control	Model	Low-dose ANP	Medium-dose ANP	High-dose ANP	Simvastatin
1	20.3 ± 0.4	21.1 ± 0.2	21.2 ± 0.3	21.6 ± 0.4	21.2 ± 0.4	21.3 ± 0.3
2	22.8 ± 0.5	23.6 ± 0.3	22.7 ± 0.4	23.3 ± 0.5	23.4 ± 0.4	23.4 ± 0.4
3	24.0 ± 0.5	25.5 ± 0.4^#^	24.7 ± 0.5	24.6 ± 0.6	24.4 ± 0.4	24.6 ± 0.4
4	25.2 ± 0.6	26.4 ± 0.5	25.5 ± 0.5	25.2 ± 0.6	25.5 ± 0.4	25.6 ± 0.4
5	25.7 ± 0.6	27.7 ± 0.5^##^	26.2 ± 0.6^*^	25.8 ± 0.7^*^	25.9 ± 0.4	26.5 ± 0.3^*^
6	27.2 ± 0.5	28.2 ± 0.4	26.9 ± 0.7	26.8 ± 0.6	26.7 ± 0.5	26.8 ± 0.5
7	27.2 ± 0.5	28.4 ± 0.5	27.2 ± 0.7	27.4 ± 0.5	27.3 ± 0.4	27.8 ± 0.4
8	28.2 ± 0.6	29.4 ± 0.5	28.0 ± 0.6	28.0 ± 0.5	28.2 ± 0.4	28.6 ± 0.4
9	28.4 ± 0.6	29.4 ± 0.5	27.8 ± 0.7^*^	28.1 ± 0.5^*^	27.8 ± 0.5^*^	27.9 ± 0.5^*^
10	28.4 ± 0.7	30.3 ± 0.5^#^	28.4 ± 0.6^*^	28.5 ± 0.5^*^	28.4 ± 0.5^*^	28.4 ± 0.6^*^

Compared with Control, ^#^p < 0.05, ^##^p < 0.01; Compared with model group, *p < 0.05, (mean ± SEM, n = 12).

### Effect of ANP on Serum CHOL, TG, LDL-C, HDL-C, and LDL-C/HDL-C Ratio

**Figure 2:**
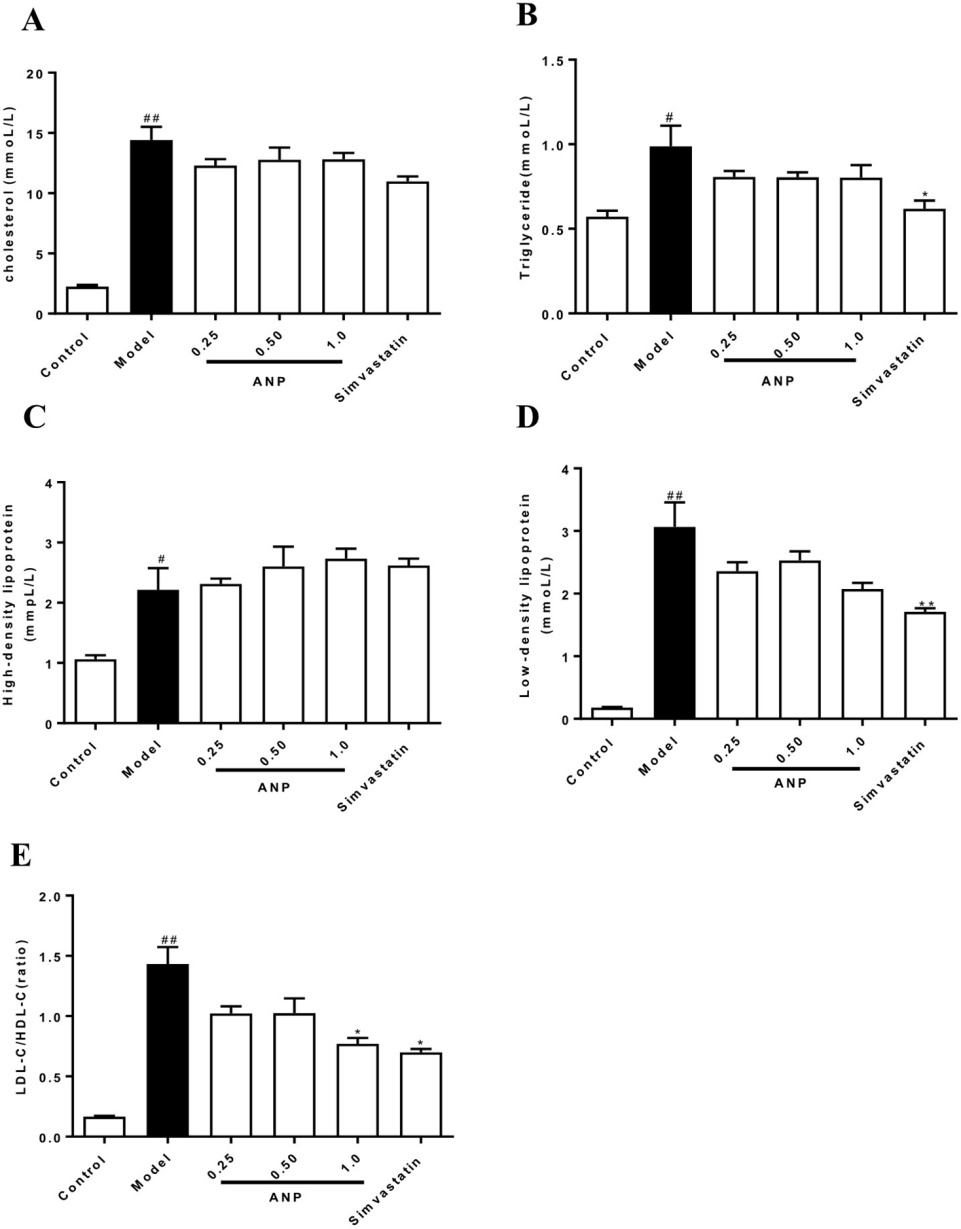
Effect of ANP on concentrations of serum CHOL, TG, HDL-C, LDL-C and LDL-C/HDL-C ratio. **(A)** Serum concentrations of CHOL, **(B)**, Serum concentrations of TG, **(C)** Serum concentrations of HDL-C, **(D)** Serum concentrations of LDL-C, **(E)** Ratio of LDL-C/HDL-C. Compared with control group, ^#^ *p* < 0.05, ^##^ *p* < 0.01; Compared with model group, *p < 0.05 (Mean ± SEM, n = 6). ANP, Angong Niuhuang Pill; CHOL, cholesterol; TG, triglyceride; LDL-C, low-density lipoprotein; HDL-C, high-density lipoprotein.

LDL-C/HDL-C ratio and concentrations of CHOL, TG, LDL-C, HDL-C in model group were higher than in control group (*p* < 0.05 or 0.01). It should be noted that all ANP administrated groups showed no difference in serum CHOL, TG, LDL-C, HDL-C. However, when compared with model group, high-dose ANP significantly decreased LDL-C/HDL-C ratio (*p* < 0.05). These results were shown in **Figures 2A–E**.

### Effect of ANP on Serum Concentrations of IL-6 and IL-10

**Figure 3:**
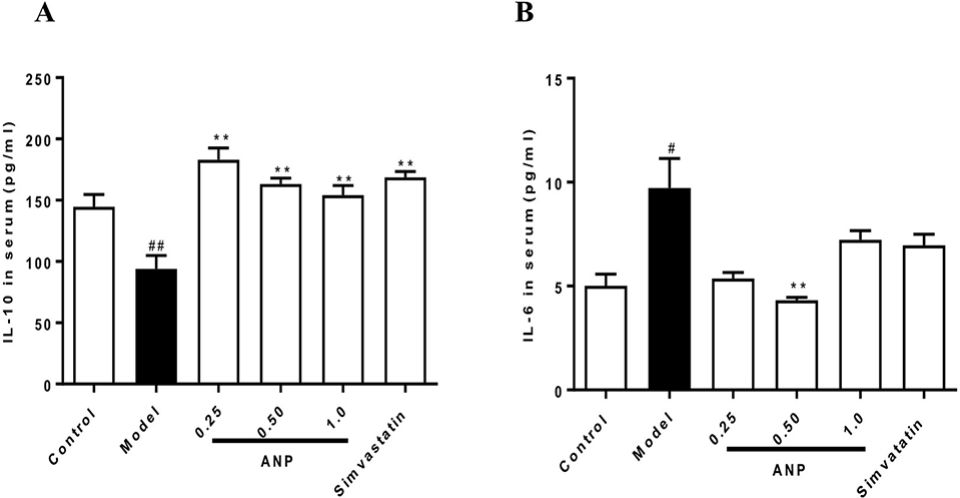
Effect of ANP on serum concentrations of pro-inflammation IL-6 and anti-inflammation IL-10. **(A)** Serum concentrations of IL-10, **(B)** Serum concentrations of IL-6. Compared with control group, ^#^ *p* < 0.05, ^##^ *p* < 0.01; Compared with model group, **p < 0.01 (Mean ± SEM, n = 6). ANP, Angong Niuhuang Pill.

Serum concentrations of IL-6 and IL-10 were shown in **Figures 3A, B**. When compared with control group, concentrations of pro-inflammation IL-6 were significantly increased (*p* < 0.05), and concentrations of anti-inflammation IL-10 were significantly decreased (*p* < 0.01). These data indicate indicated that ApoE^-/-^ mice of model group had a chronic inflammation at the early and mid-term AS. When compared with model group, medium-dose ANP significantly decreased concentrations of serum IL-6 (*p* < 0.01), and all ANP administrated groups significantly increased concentrations of serum IL-10.

### Effect of ANP on Th17/Treg Balance in the Spleen

**Figure 4:**
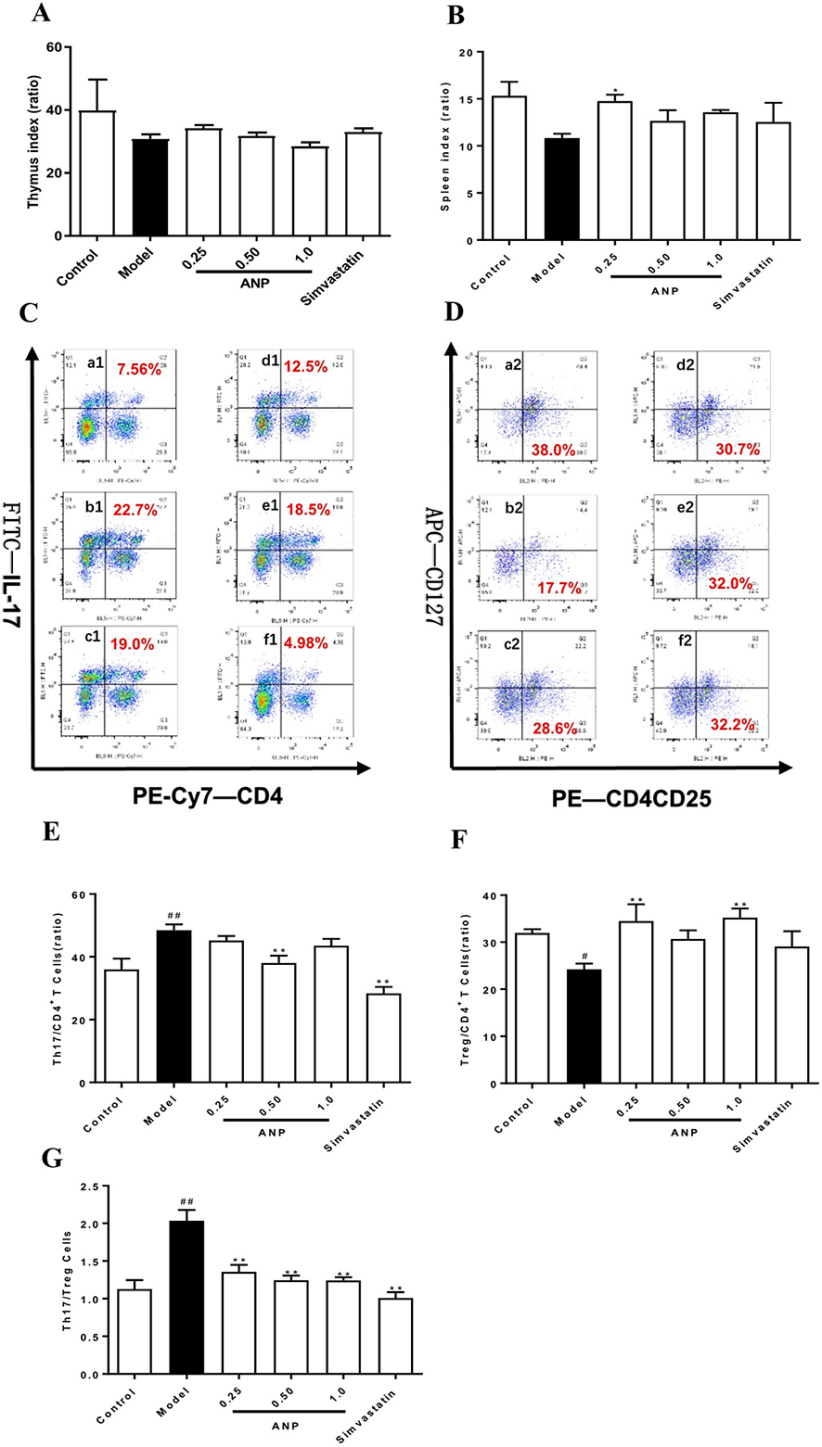
Effect of ANP on Th17/Treg balance in the spleen. **(A)** Thymus index, **(B)** Spleen index, **(C)** Analysis of Th17 cells, **(D)** Analysis of Treg cells, **(E)** Th17/CD4+ T cells, **(F)** Treg/CD4+ T cells, **(G)** Th17/Treg cells. a1 and a2: Control group, b1 and b2: Model group, c1 and c2: Low-dose ANP group, d1 and d2: Medium-dose ANP group, e1 and e2: High-dose ANP group, f1 and f2: Simvastatin group. Compared with control group, ^#^ *p* < 0.05, ^##^ *p* < 0.01; Compared with model group, **p < 0.01 (Mean ± SEM, n = 6). ANP, Angong Niuhuang Pill.

According to results of thymus index and spleen index (**Figures 4A, B**), there was an upward trend in the ANP groups, and low-dose ANP significantly increased spleen index (*p* < 0.05). It suggests that ANP may affect the immune regulation of the spleen. In order to verify above results, splenocytes were used to analyze Th17/Treg balance in the spleen by flow cytometric analysis. In the model group, Th17/CD4^+^T cells ratio, and Th17/Treg ratio were significantly increased, however, Treg/CD4^+^T cells ratio was significantly decreased (*p* < 0.05 or 0.01). After ANP administration, when compared with model group, it was shown that medium-dose ANP significantly decreased Th17/CD4^+^T cells ratio (*p* < 0.01), while low-dose and high-dose ANP significantly increased Treg/CD4^+^T cells ratio (*p* < 0.01). It was also shown that all ANP administrated groups could decrease Th17/Treg ratio (**Figures 4C–G**).

### Effect of ANP on Expression of RORγt and Foxp3 in the Spleen

**Figure 5:**
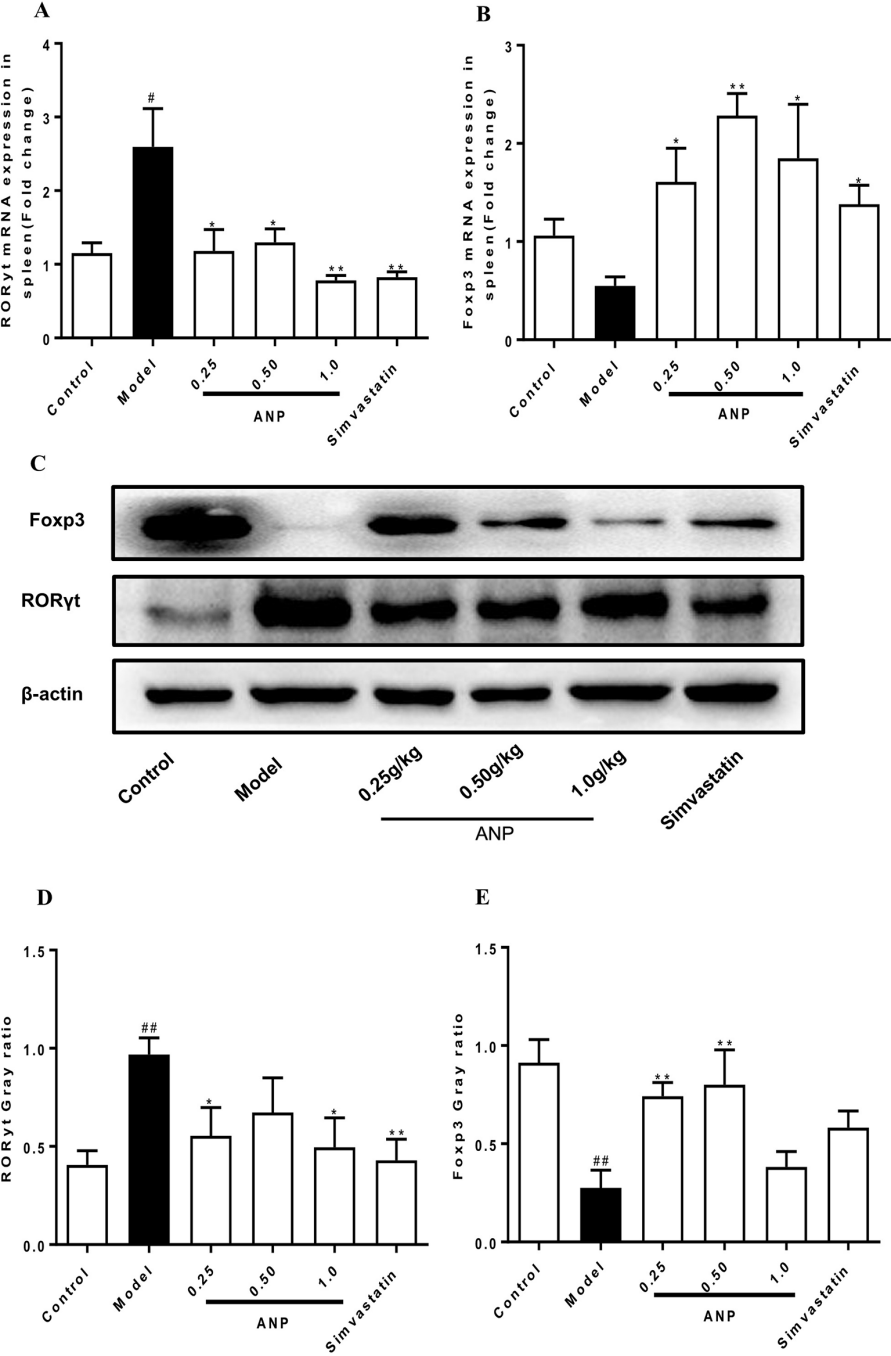
Effect of ANP on expression of RORγt and Foxp3 in the spleen. **(A)** mRNA expression levels of RORγt, **(B)** mRNA expression levels of Foxp3, **(C)** Western blotting analysis of RORγt and Foxp3. The original blot image of Foxp3 was shown in **Supplementary Figure S4(a)**. **(D)** Semi-quantitative analysis of RORγt protein, **(E)** Semi-quantitative analysis of Foxp3 protein. Compared with control group, ^#^ *p* < 0.05, ^##^ *p* < 0.01; Compared with model group, *p < 0.05, **p < 0.01 (Mean ± SEM, RT-qPCR experiment n = 6; Western blotting experiment n = 3). ANP, Angong Niuhuang Pill.

RORγt and Foxp3 are transcription factors for Th17 cells which regulate Th17/Treg balance. To evaluate effect of ANP on Th17/Treg balance, expression levels of RORγt, and Foxp3 were detected in the spleen. In the model group, mRNA and protein expression levels of RORγt was significantly up-regulated (*p* < 0.05 or 0.01), and Foxp3 was significantly down-regulated (*p* < 0.05 or 0.01). After ANP administration, when compared with model group, it was shown that low-dose and high-dose ANP significantly down-regulated protein expression of RORγt (*p* < 0.05). Moreover, low- and medium-dose ANP significantly up-regulated protein expression of Foxp3 (*p* < 0.05 or 0.01). Similarly, in all ANP groups mRNA expression levels of RORγt were down-regulated, while mRNA for Foxp3 was up-regulated (*p* < 0.05 or 0.01; **Figures 5A–E**).

### Effect of ANP on Expression of Cytokines and Chemokines in the Aorta

**Figure 6:**
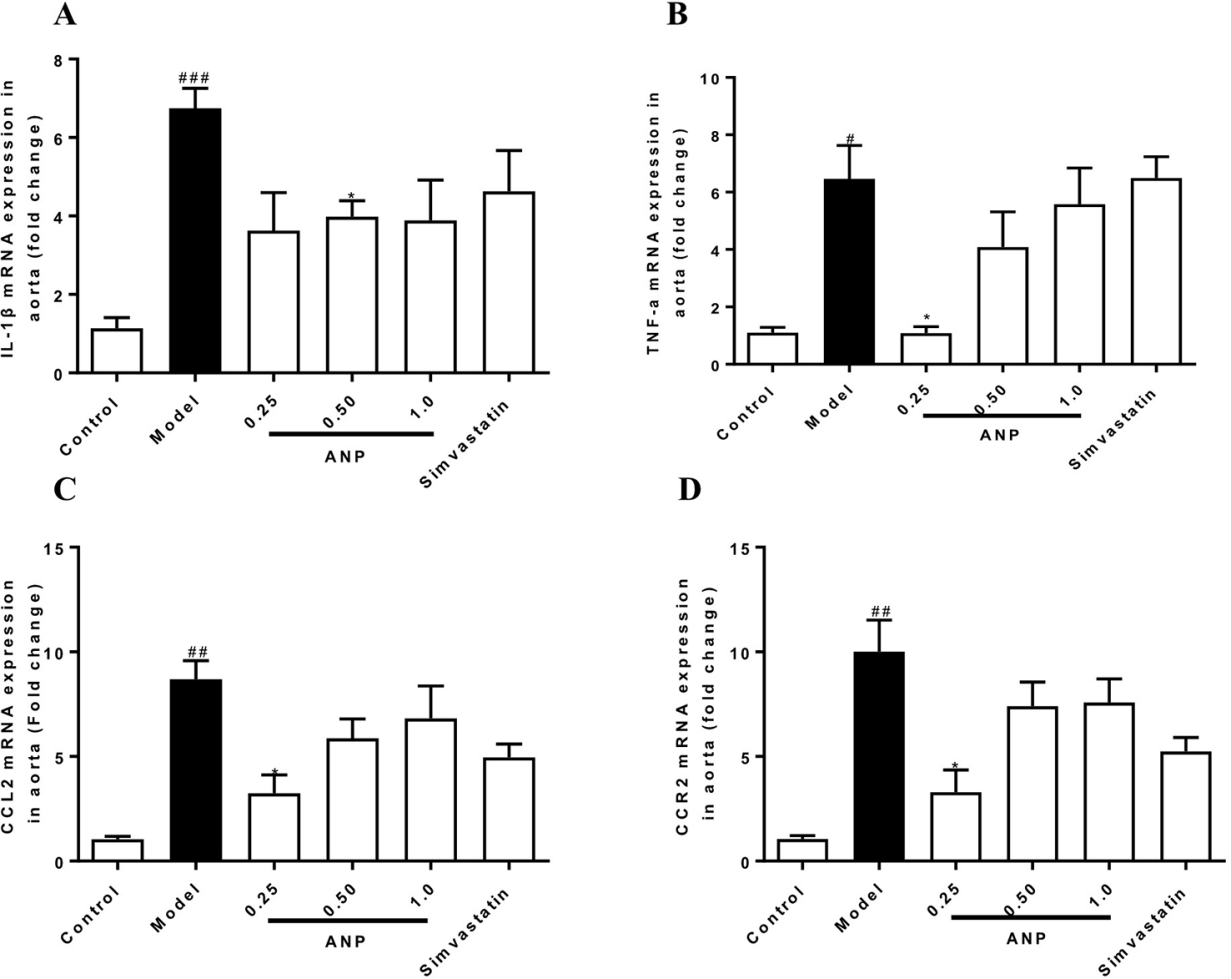
Effect of ANP on mRNA expression levels of IL-1β, TNF-α, CCL2, and CCR2 in the aorta. **(A)** mRNA expression levels of IL-1β, **(B)** mRNA expression levels of TNF-α, **(C)** mRNA expression levels of CCL2, **(D)** mRNA expression levels of CCR2. Compared with control group, ^#^ *p* < 0.05, ^##^ *p* < 0.01, ^###^ *p* < 0.001; Compared with model group, *p < 0.05. (Mean ± SEM, n = 6). ANP, Angong Niuhuang Pill.

**Figure 7:**
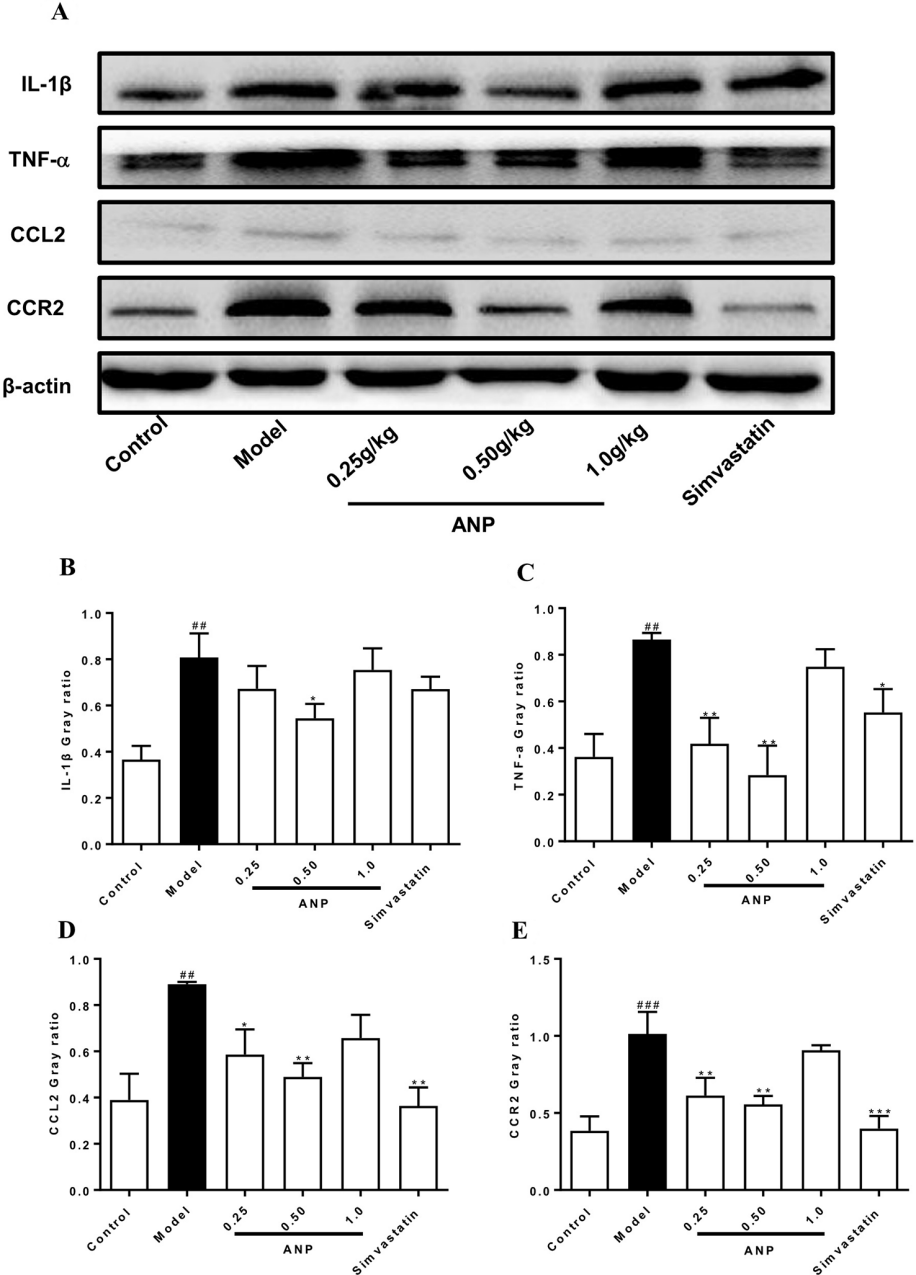
Effect of ANP on protein expression of IL-1β, TNF-α, CCL2 and CCR2 in the aorta. **(A)** Western blotting analysis of IL-1β, TNF-α, CCL2, and CCR2. **(B)** Semi-quantitative analysis of IL-1β protein. The original blot images of IL-1β, TNF-α were shown in **Supplementary Figure S4(b)** and **Figure S4(c)**. **(C)** Semi-quantitative analysis of TNF-α protein, **(D)** Semi-quantitative analysis of CCL2 protein, **(E)** Semi-quantitative analysis of CCR2 protein. Compared with control group, ^##^ *p* < 0.01, ^###^ *p* < 0.001; Compared with model group, *p < 0.05, **p < 0.01, ***p < 0.001 (Mean ± SEM, n = 3). ANP, Angong Niuhuang Pill.

Cytokines and chemokines play important roles in early and mid-term of AS and have a profound influence on the pathogenesis of AS. When compared to the control group, mRNA and protein expression levels of IL-1β, TNF-α, CCL2, CCR2 in the model group were significantly up-regulated (*p* < 0.05 or 0.01 or 0.001). In the medium-dose ANP mRNA and protein expression levels of IL-1β were significantly down-regulated (*p* < 0.05), while in low- and medium-dose ANP mRNA and protein expression levels of TNF-α, CCL2, and CCR2 were also significantly down-regulated (*p* < 0.05 or 0.01). At the mRNA level, the anti-inflammatory effect of the ANP was better than the positive drug simvastatin. It is worth noting that low- and medium-dose ANP had better anti-inflammatory effect, but there was no significantly difference in the high-dose ANP group. These results were shown in **Figures 6A–D** and **Figures 7A–E**.

### Effect of ANP on Expression of MMP-2 and MMP-9 in the Aorta

**Figure 8:**
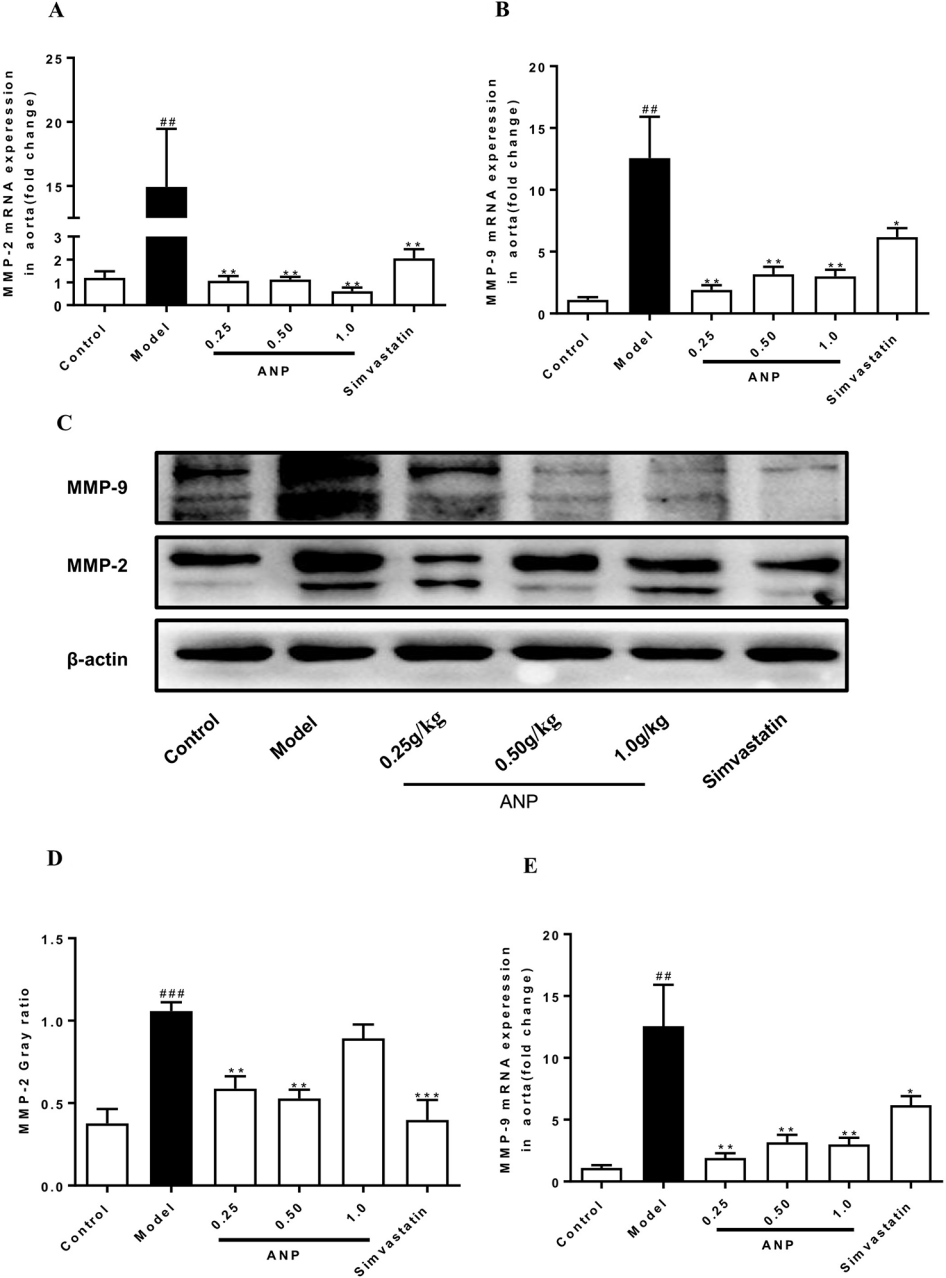
Effect of ANP on mRNA and protein expression of MMP-2 and MMP-9 in the aorta. **(A)** mRNA expression levels of MMP-2, **(B)** mRNA expression levels of MMP-9, **(C)** Western blotting analysis of MMP-2 and MMP-9, **(D)** Semi-quantitative analysis of MMP-2 protein, **(E)** Semi-quantitative analysis of MMP-9 protein. Compared with control group, ^##^ *p* < 0.01, ^###^ *p* < 0.001; Compared with model group, *p < 0.05, **p < 0.01, ***p < 0.001 (Mean ± SEM, RT-qPCR experiment n = 6; Western blotting experiment n = 3). ANP, Angong Niuhuang Pill.

MMPs are a group of proteases that degrade extracellular matrix, and they are mainly secreted by macrophages and VSMCs in atherosclerotic plaques. Overexpression of MMPs promotes the degradation of collagen and elastic fibers in plaque, destroying the stability of arterial plaque, which facilitate induction of cardiovascular risk events. As shown in **Figures 8 A–E**, both mRNA and protein expression levels of MMP-2 and MMP-9 in the model group were significantly up-regulated (*p* < 0.01 or 0.001). Administration of ANP significantly down-regulated mRNA and protein expression levels of MMP-2 and MMP-9 (*p* < 0.05 or 0.01).

### Effect of ANP on Collagen Fibers in the Aortic Root

**Figure 9:**
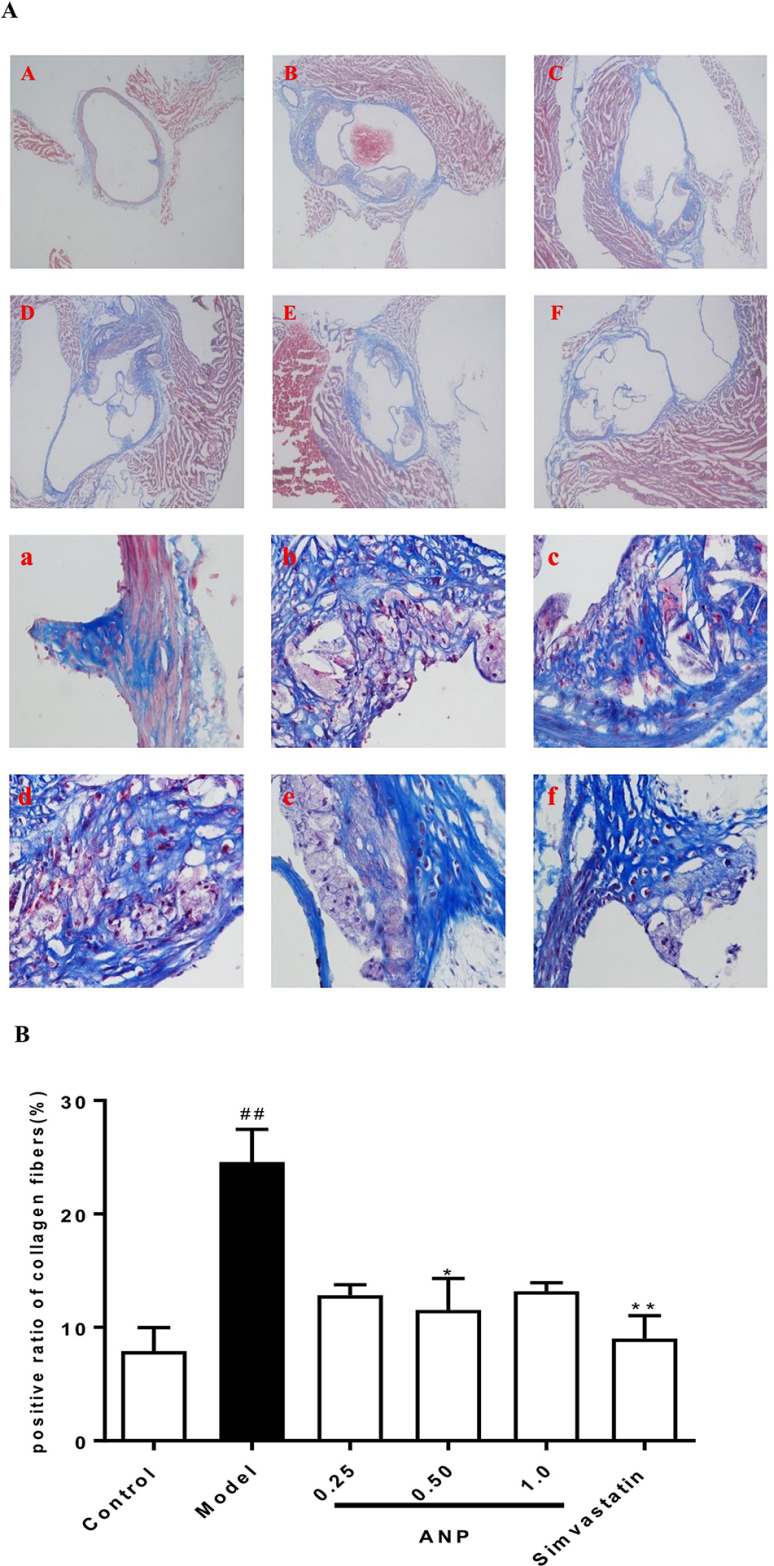
Effect of ANP on collagen fibers in the aortic root. **(A)** Masson staining was performed in the aortic root, A and a: Control group, B and b: Model group, C and c: Low-dose ANP group, D and d: Medium-dose ANP group, E and e: High-dose ANP group, F and f: Simvastatin group. Picture A B C D E F ×40, Picture a b c d e f ×400. **(B)** Semi-quantitative analysis of collagen fibers. Compared with control group, ^##^ *p* < 0.01; compared with model group, *p < 0.05, **p < 0.01 (Mean ± SEM, n = 6). ANP, Angong Niuhuang Pill.

Masson staining was performed in the aortic root. The area of fibrosis which was stained blue covered a much larger area in the model group than in the control group (*p* < 0.01). Conversely, the area of collagen fiber in the medium-dose ANP were significantly lower than the model group (*p* < 0.05). These results were shown in **Figures 9A, B**.

### Effect of ANP on Inflammatory Cells Infiltration in the Aortic Root

**Figure 10:**
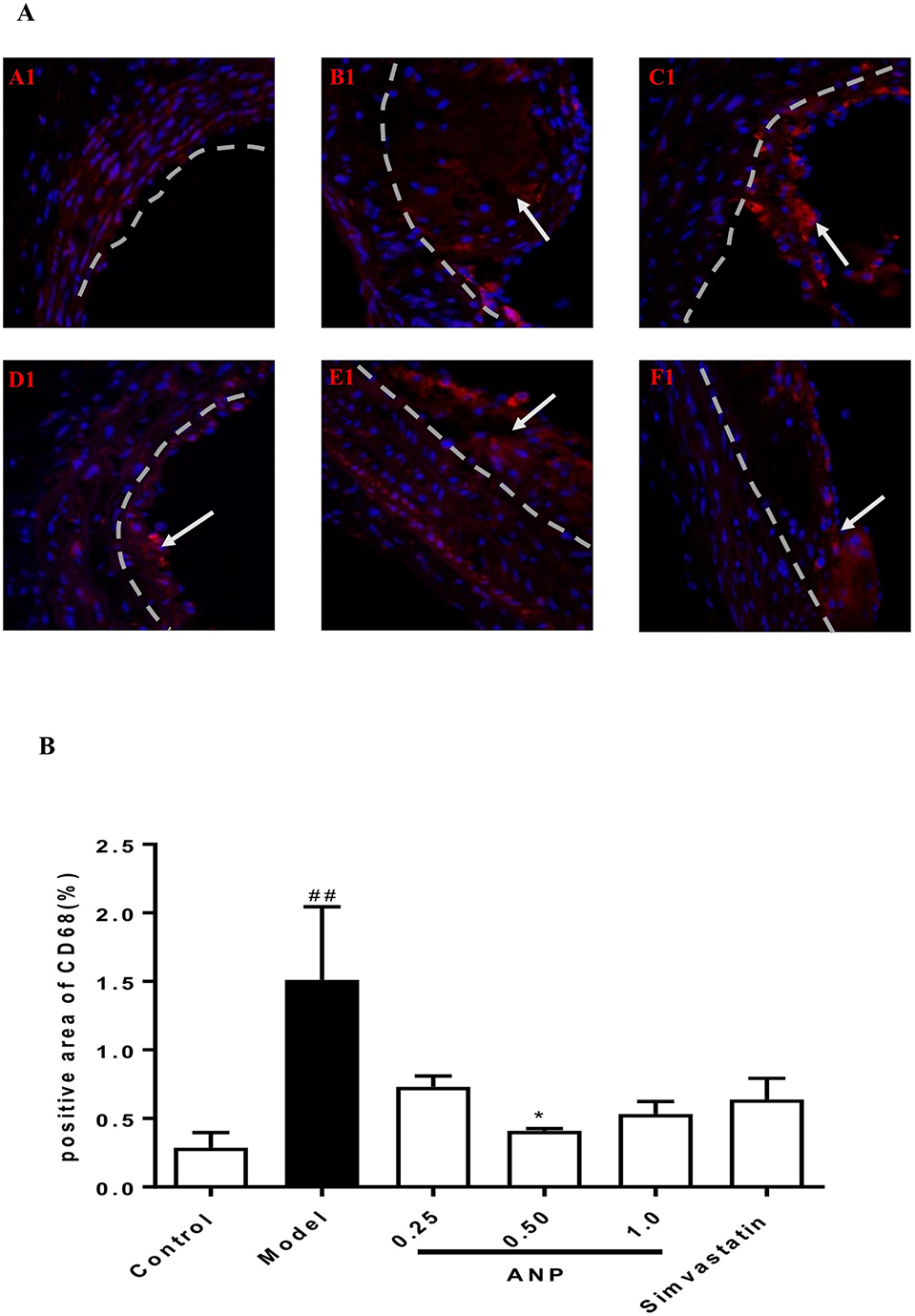
Effect of ANP on macrophages infiltration in the aortic root. **(A)** Immunofluorescence staining of macrophages, white arrow represents the positive area of macrophages. A1: Control group, B1: Model group, C1: Low-dose ANP group, D1: Medium-dose ANP group, E1: High-dose ANP group, F1: Simvastatin (Picture A1 B1 C1 D1 E1 F1×400). **(B)** Semi-quantitative analysis of macrophages, positive area of CD68 for macrophages. Compared with control group, ^##^ *p* < 0.01; compared with model group, *p < 0.05 (Mean ± SEM, n = 3). ANP, Angong Niuhuang Pill.

**Figure 11:**
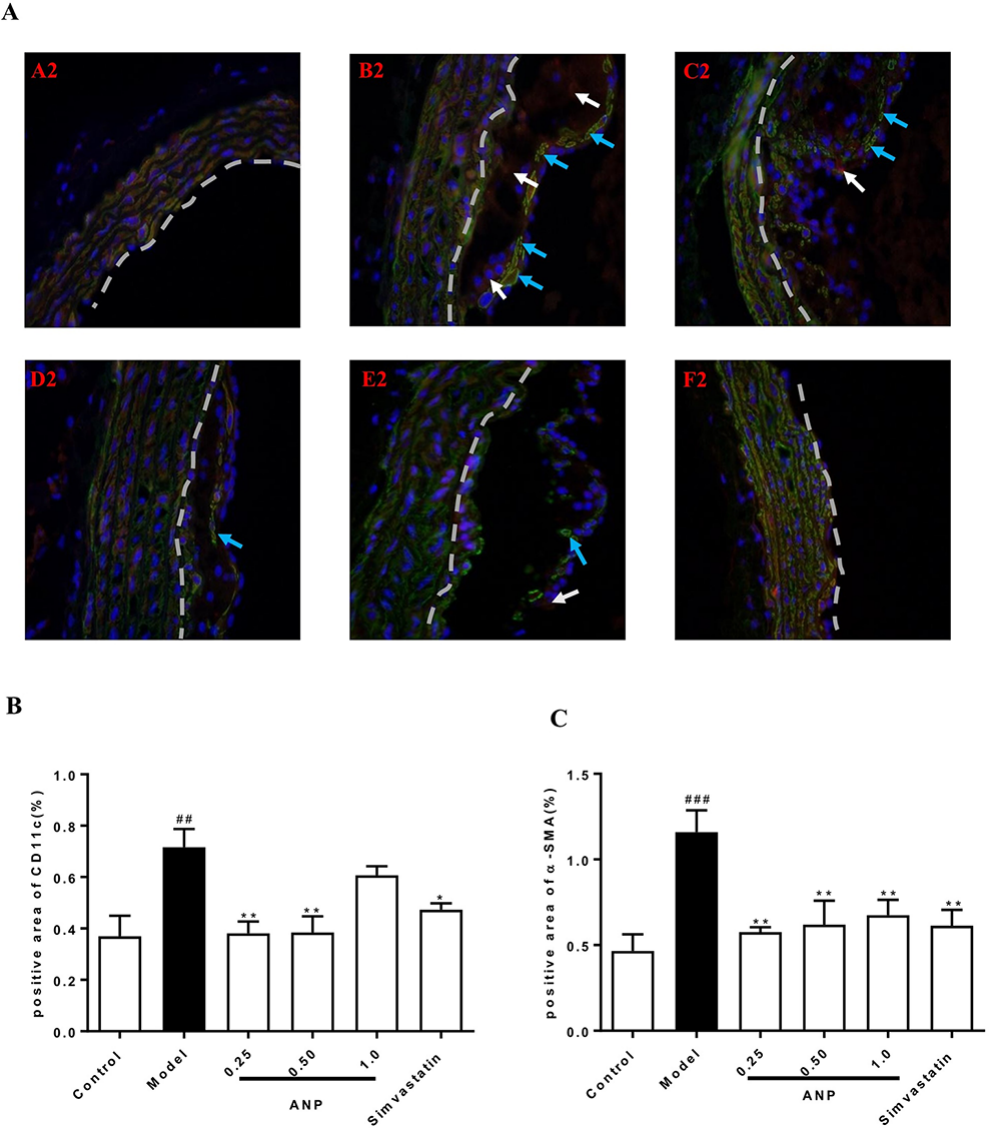
Effect of ANP on VSMCs and dendritic cells infiltration in the aortic root. **(A)** Immunofluorescence staining of VSMCs and dendritic cells, white arrow represents the positive area of dendritic cells, and blue arrow represents the positive area of VSMCs. A2: Control group, B2: Model group, C2: Low-dose ANP group, D2: Medium-dose ANP group, E2: High-dose ANP group, F2: Simvastatin (Picture A2 B2 C2 D2 E2 F2×400), **(B)** Semi-quantitative analysis of dendritic cells, positive area of CD11c for dendritic cells, **(C)** Semi-quantitative analysis of VSMCs, positive area of α-SMA for VSMCs. Compared with control group, ^##^ *p* < 0.01, ^###^ *p* < 0.001; compared with model group, *p < 0.05, **p < 0.01 (Mean±SEM, n = 3). ANP, Angong Niuhuang Pill.

Immunofluorescence staining was performed in the aortic root. In the model group, the positive area of macrophages which were stained red was higher than in the control group (*p* < 0.01), and the positive area of VSMCs and dendritic cells, which was respectively stained green and red also was higher than that in the model group (*p* < 0.001). In low, or medium or high-dose ANP groups, the positive area of these inflammatory cells was significantly lower than in the model group (*p* < 0.01 or 0.001). These results were shown in **Figures 10A, B** and **Figures 11A–C**.

## Discussion

We found that treatment with ANP corrected the immune imbalance of Th17/Treg cells in the early AS ApoE^-/-^ mice model by regulating RORγt and Foxp3 expression; ANP suppressed the release of pro-inflammatory mediators, promoted the release of anti-inflammatory factors, down-regulated the expression of chemokines and their receptors promoting thus an anti- inflammatory effects in the early AS. Furthermore, ANP down-regulated expression of MMP-2, MMP-9, reduce collagen fibers and decreased infiltration of macrophages, dendritic cells, and vascular smooth muscle cells into plaques thus facilitating stability of the latter.

In this study, ANP was a kind of tawny powder, and distilled water was used as the solution medium. Due to the relatively complex composition of ANP, ANP was tawny suspension after dissolution. In order to maintain the stability of drug suspension, the ANP suspension was prepared before administration. In our next study, we will focus on the metabolic components of ANP in mice and the pharmacodynamic substance basis of ANP, and analyze the prescription.

It has been argued that increase in combined ratios (LDL-C/HDL-C) relates to the severity and prevalence of coronary artery disease (Enomoto et al., 2011; Acay et al., 2014). We demonstrated that LDL-C/HDL-C ratio in the high-dose ANP group (1.0 g/kg) was significantly lower than those in the model group. However, low-dose and medium-dose ANP groups did not show a decrease in LDL-C/HDL-C ratio, which indicates that only a high-dose ANP has a weak lipid-lowering effect.

The high-fat diet is a potential risk factor for the liver and kidney. Treatment with ANP significantly reduced the liver coefficient but did not improve the kidney coefficient; thus pointing on a certain hepatoprotective effect of ANP. This agreed with previous findings showing that ANP has a preventive and therapeutic effect on liver damage caused by bacterial toxins (He et al., 1992). The apparent changes of spleen index and thymus index suggest that ANP may have a regulatory effect on the immune system. The Th17/Treg is a pair of new balance which differs from Th1/Th2 balance in the immune system and plays an important role in the development of AS and plague rupture (Cheng et al., 2008; Xie et al., 2010). Many studies have confirmed that regulating Th17/Treg balance is a critical intervention target during development process of AS (Xie et al., 2010; Konstantinos et al., 2014; Tian et al., 2017; Wang et al., 2017). Treatment with ANP corrected the Th17/Treg cellular balance, with high doses of ANP having more pronounced immunomodulatory effect. The Th17/Treg balance is regulated by RORγt and Foxp3 respective transcription factors (Xie et al., 2010). We found that ANP significantly down-regulated expression of RORγt, while significantly up-regulating expression of Foxp3; indicating the underlying mechanisms.

Chronic inflammation is the key factor in AS pathogenesis, while inhibiting inflammatory response controls AS evolution (Tousoulis et al., 2016; Chistiakov et al., 2018). Inflammatory cells involved in the AS process can express different inflammatory factors, such as IL-1β, TNF-α, and IL-6, which are mainly secreted by macrophages, lymphocytes, and VSMCs (Tedgui and Mallat, 2006). Expression of IL-1β and TNF-α is regulated by the p38 MAPK/NF-κB pathway (Chan et al., 2000). Increased expression of IL-1β and TNF-α promotes overexpression of downstream ICAM-1 and VCAM-1, to further promote migration and adhesion of VSMCs and endothelial cells (Mackesy and Goalstone, 2014). Expression of IL-6 is regulated by IL-6 receptor and signal transducing protein gp130 (Ait-Oufella et al., 2011); IL-6 being a pro-atherogenic cytokine exacerbates the progression of atherosclerosis in ApoE^-/-^ mice. Clinical studies revealed that high levels of serum IL-6 represent a risk factor for coronary artery disease (Biasucci, 1999). The anti-atherogenic cytokine IL-10 is secreted by Treg cells; IL-10 not only down-regulates expression of TNF-α, but also reduces expression of ICAM-1 in endothelial cells (Lisinski and Furie, 2002; Rajasingh et al., 2006). Treatment with ANP significantly reduced serum IL-6 and increased serum IL-10, although the dose-dependence was not obvious. Expression of TNF-α was significantly down-regulated in low-dose and medium-dose ANP groups, and expression of IL-1β was significantly down-regulated in low-dose ANP group. Thus ANP (especially at low and medium doses) exhibits anti-inflammatory capabilities.

Atherosclerotic plaque, the morphological substrate of AS, is induced by multiple factors, including accumulation of VSMCs, macrophages and T lymphocytes (Andrés et al., 2012; Moore et al., 2013); the proliferation of VSMCs and connective tissue matrix such as collagen fibers and elastic fibers; deposition of cholesterol crystals and free cholesterol. All these factors contribute to formation of fragile arterial plaques (Watson et al., 2018). Maintaining stability of plaques and arresting expansion of vulnerable plaques are in the focus of current treatment for AS which has already formed. It was reported that CCL2, CCR2, MMP-2, and MMP-9 are involved in impairing stability of arterial plaques, reduction in expression of plaques stability (Ishibashi et al., 2004; Krohn et al., 2007; Zhao, 2010; Cipriani et al., 2013). We found that ANP significantly down-regulated expression of CCL2, CCR2, MMP-2, and MMP-9. Masson staining showed that medium-dose ANP decreased plaques area in the aortic root when compared to the model group, similarly reduced was the content of collagen fibers. Treatment with ANP also reduced infiltration of macrophages, vascular smooth muscle cells and dendritic cells into the aortic root as evidenced by immunocytochemical analysis. These results indicate that ANP may improve the stability of arterial plaques and prevent their expansion, suppressing infiltration of inflammatory cells, and decreasing expression of CCL2, CCR2, MMP-2, and MMP-9. All these findings suggest that, ANP may exert an anti-atherosclerotic effect at the early and mid-term of AS.

## Conclusion

**Figure 12:**
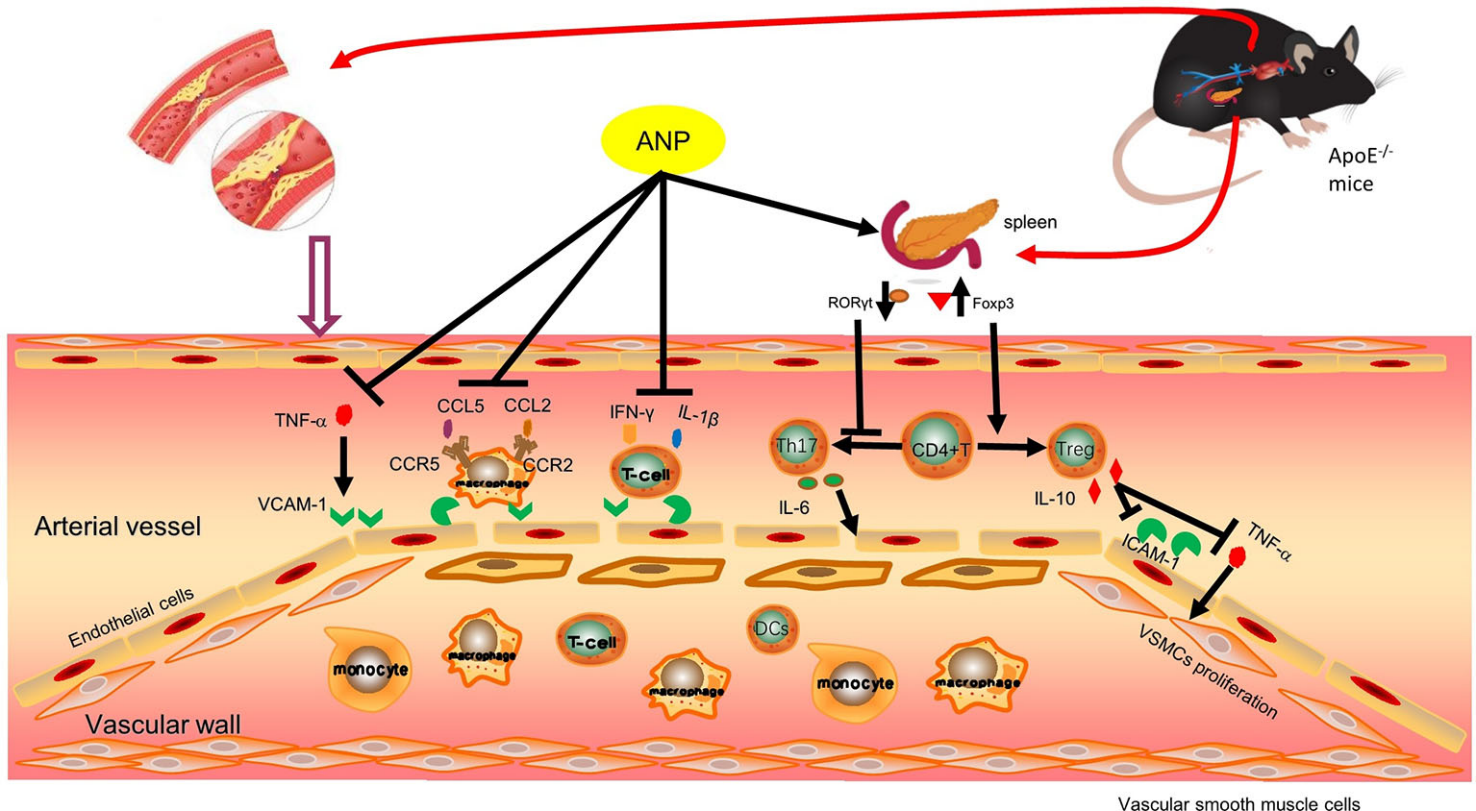
Possible mechanism pathway of ANP. Previous research findings found that ANP could down-regulated mRNA expression levels of IFN-γ, CCL5 and CCR5, these results were shown in **Supplementary Material** (**Figure S2**). ANP, Angong Niuhuang Pill.

In conclusion, ANP protects early and mid-term atherosclerotic ApoE^-/-^ mice by regulating Th17/Treg balance, inhibiting chronic inflammation, reducing plaque collagen fibers, and reducing inflammatory cells infiltration. Moreover, ANP has anti-inflammatory effects in small doses, and ANP has immunoregulation effects on high doses. Possible mechanisms are shown in **Figure 12**.

## Data Availability Statement

All datasets generated for this study are included in the article/**Supplementary Material**.

## Ethics Statement

The experiments were approved by the Laboratory Animal Ethics Committee of Jinan University (No. 201812374), and were performed according to the instructions of the National Institute of Health (OLAW/NIH Revised 2015).

## Author Contributions

HN supported and designed the experiments, and revised the manuscript. QF performed the experiments. YL, JR, ZZ, KY, WX, TZ, XC, NN, ZY, YC, and YX provided reagents/materials. YL and JR analyzed the data. RL participated in checking data statistics and manuscript revision linguistically. AV gave guidance during the experimental design process, and revised the manuscript. QF and YL wrote the manuscript.

## Funding

This work was supported by the National Natural Science Foundation of China (No. 81673634) and Guangzhou Baiyunshan Zhongyi Pharmaceutical Co., Ltd (No. 40116103).

## Conflict of Interest

Authors NN, ZY, YC and YX are employed by company Guangzhou Baiyunshan Zhongyi Pharmaceutical Co., Ltd. Author RL is a student of International Department, the Affiliated High School of SCNU. Author AV is a professor in the Faculty of Biology, Medicine and Health, The University of Manchester.

The authors declare that this study received funding from Guangzhou Baiyunshan Zhongyi Pharmaceutical Co., Ltd. The funders had no role in study design, data collection and analysis, decision to publish and preparation of the manuscript.

The remaining authors declare that the research was conducted in the absence of any commercial or financial relationships that could be construed as a potential conflict of interest.
