# Supplementary material for: Anti-Atherosclerosis Effect of Angong Niuhuang Pill via Regulating Th17/Treg Immune Balance and Inhibiting Chronic Inflammatory on ApoE-/- Mice Model of Early and Mid-Term Atherosclerosis
Source: Front Pharmacol. 2020 Jan 31;10:1584. doi: 10.3389/fphar.2019.01584 (PMC7005527; doi:10.3389/fphar.2019.01584)
Supplement: Supplementary file 1 [file DataSheet_1.pdf]

Supplementary Material

1. Supplementary Figures

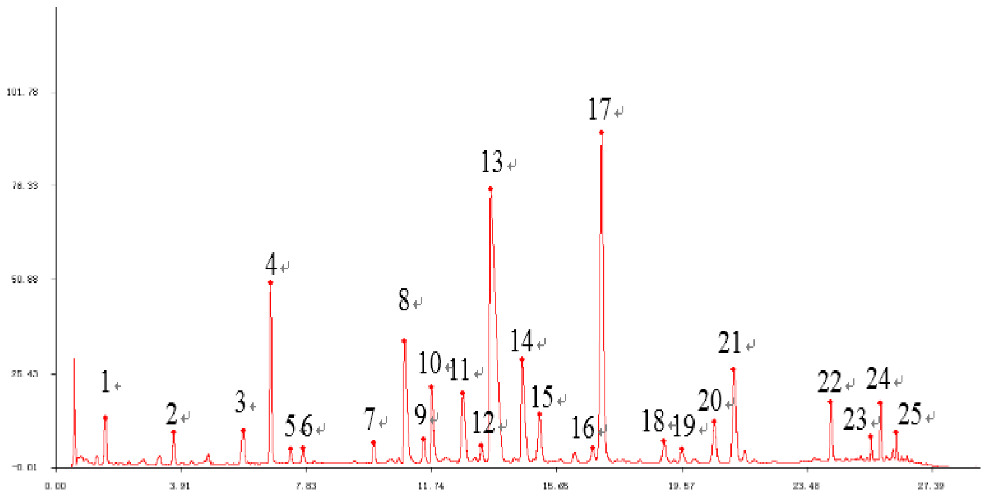

FigureS1. The fingerprint chromatography of ANP(peak 2: p-hydroxybenzonic acid, from Moschus; peak 3: Genipin-1-β-D-gentiobioside, from Gardenia jasminoides Ellis; peak 4: Geniposide, from Gardenia jasminoides Ellis; peak 7: Groenlandicine, From *Coptis chinensis Franch*; peak 8: Coptisine, From *Coptis chinensis Franch*; peak 10: Epiberberine, From *Coptis chinensis Franch*; peak 11: Jatrorrhizine, From *Coptis chinensis Franch;* peak 13: Berberine, From *Coptis chinensis Franch;* peak 14: Palmatine, From *Coptis chinensis Franch;* peak15: Ethyl 4-hydroxybenzoate,From Moschus; peak 17: Baicalin, From *Scutellaria baicalensis Georgi;* peak 19: Chrysin-7-O-Beta-D-glucoronide, From *Scutellaria baicalensis Georgi;* peak 20: Oroxyloside, From *Scutellaria baicalensis Georgi;* peak 21: Wogonoside, From *Scutellaria baicalensis Georgi;* peak 22: Baicalein, From *Scutellaria baicalensis Georgi;* peak 24: Wogonin, From *Scutellaria baicalensis Georgi;* peak 25: Oroxylin A, From *Scutellaria baicalensis Georgi*).

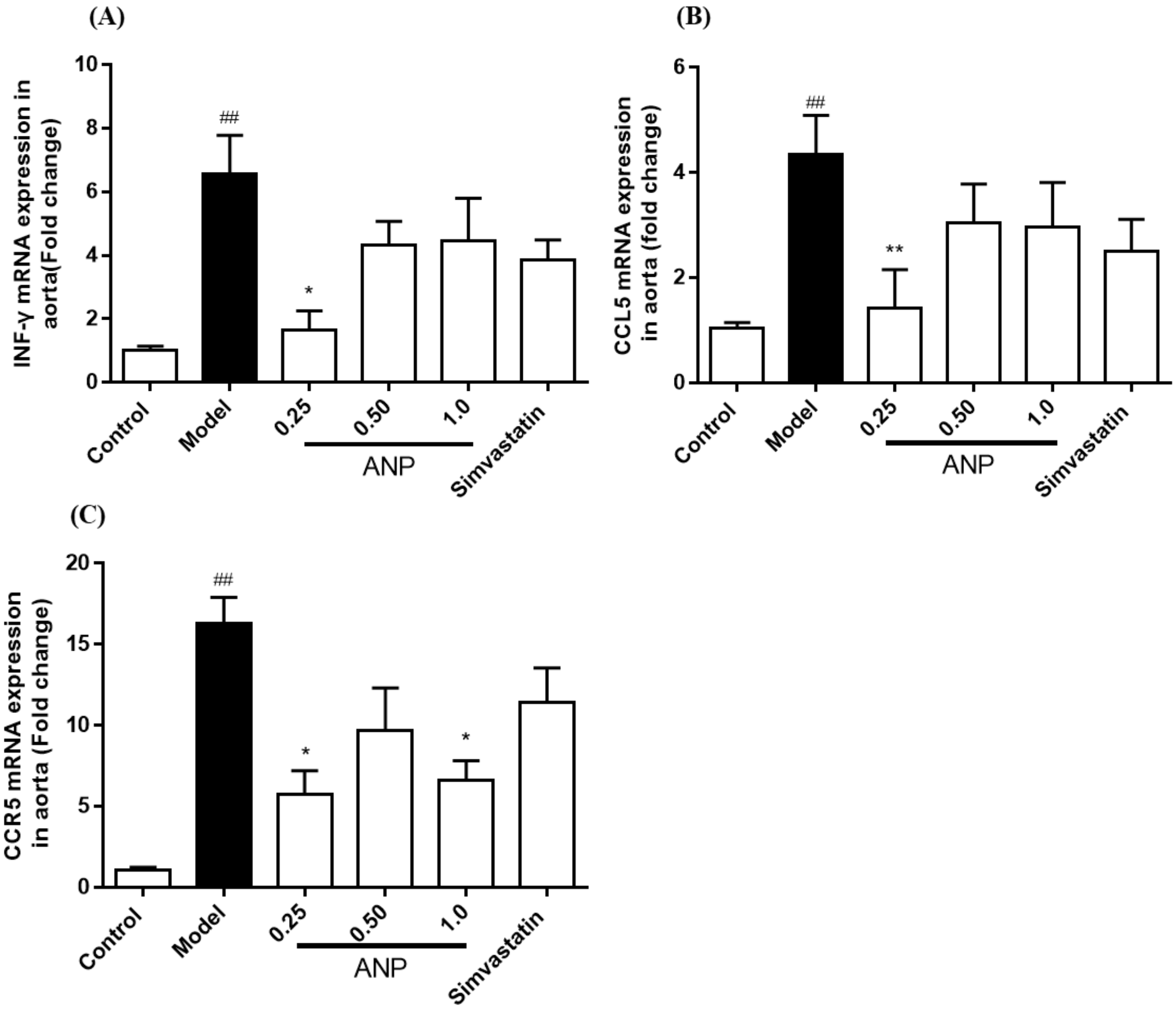

Figure S2. Effect of ANP on mRNA expression levels of IFN-γ, CCL5 and CCR5. (A): mRNA expression levels of INF-γ. (B): mRNA expression levels of CCL5. (C): mRNA expression levels of CCR5. Compared with control group, $^{\#\#}p < 0.01$; compared with model group, $^{*}p < 0.05$, $^{**}p < 0.01$ (Mean±SEM, n=6).

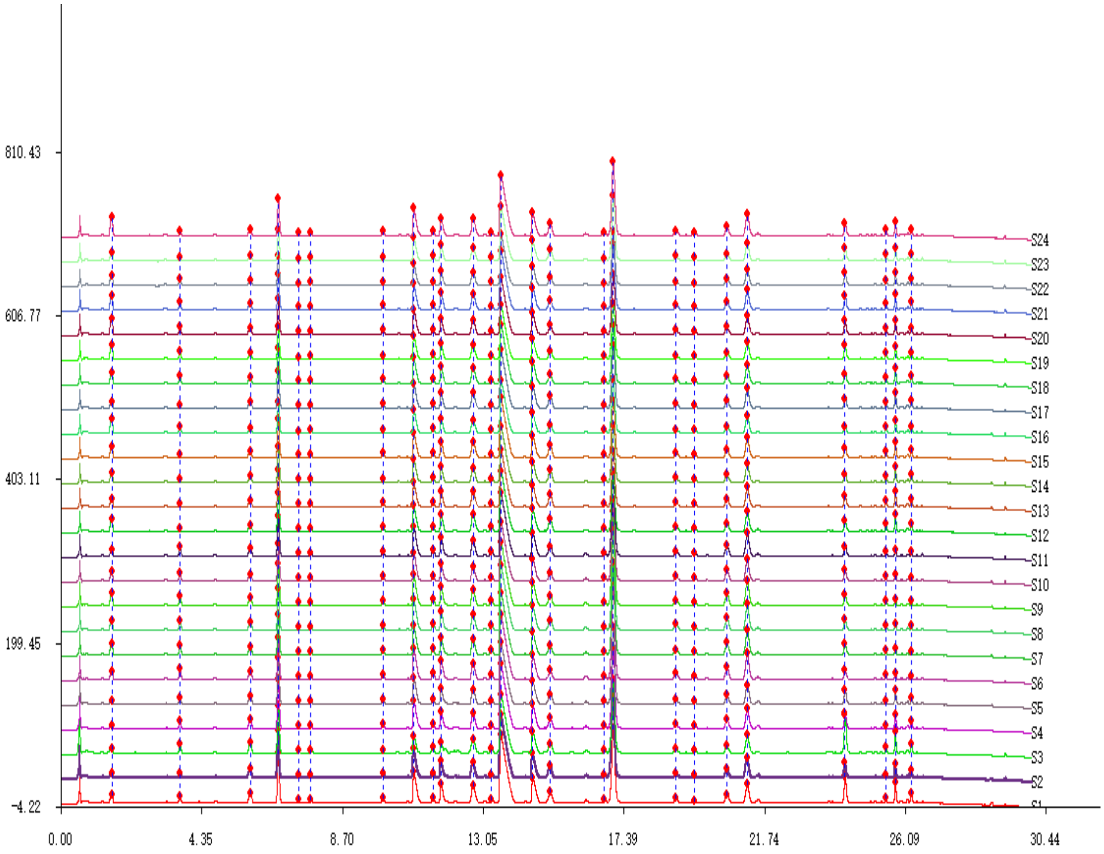

FigureS3. The UPLC Fingerprint of 24 ANP Batches

(a)

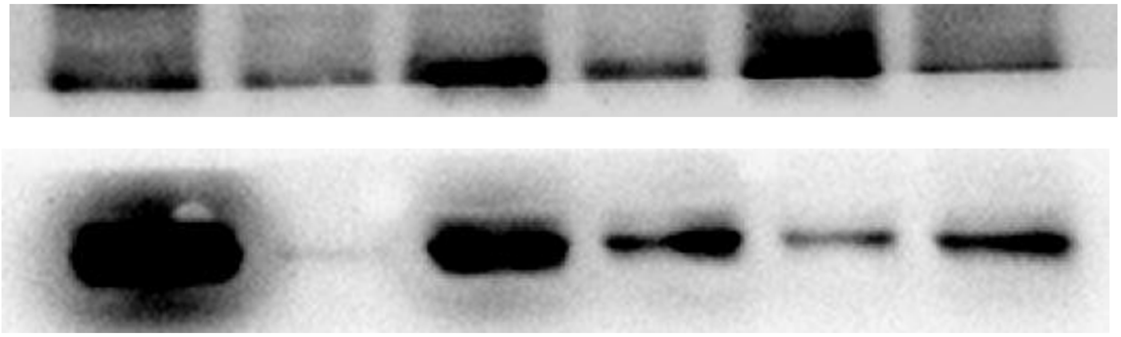

(b)

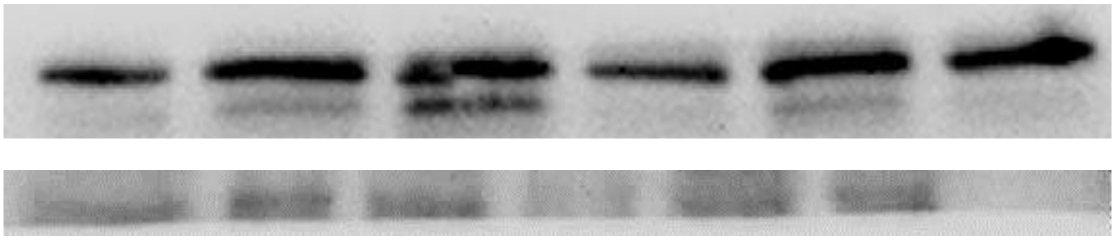

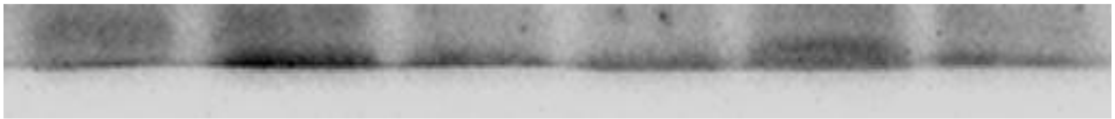

(c)

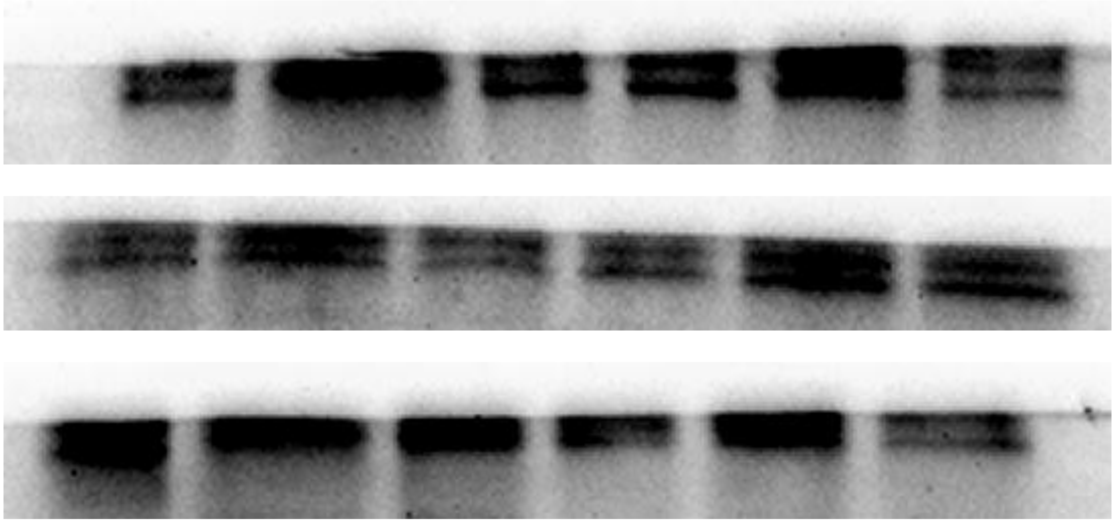

FigureS4. The original blot images of Foxp3(a), IL-1β(b), TNF-α(c).

2. Supplementary Tables

Table S1. The chemical structure of the known component in artificial Moschus

No	Compound	Chemical structure
1	cyclopentadecanone,3-methyl-	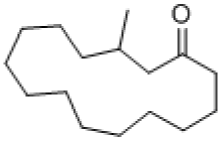
2	（3β）-cholest-5-en-3-ol	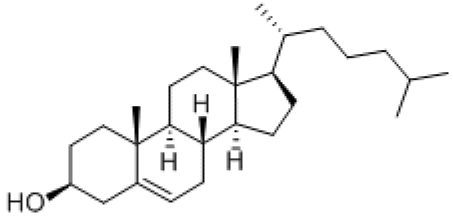
3	oleic acid	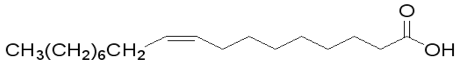
4	prasterone	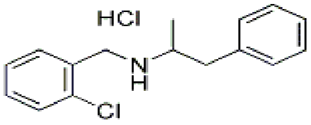
5	octadecanoic acid	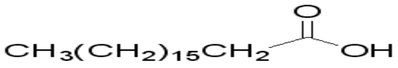
6	tetradecanoic acid	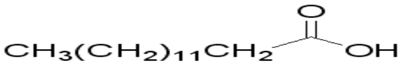
